# Attitudes and Preferences Towards Screening for Dementia with a Focus on Ethnic Minority and Low Socio-Economic Groups: A Systematic Review of Research Studies Written in the English Language

**DOI:** 10.3233/JAD-240315

**Published:** 2024-08-13

**Authors:** Manjot Brar, Ríona Mc Ardle, Alexander Hagan, Amani Al-Oraibi, Matilda Hanjari, Blossom Stephan, Carol Brayne, Louise Lafortune, Manpreet Bains, Nadeem Qureshi, Louise Robinson

**Affiliations:** a Population Health Sciences Institute, Newcastle University, Newcastle upon Tyne, UK; b Translational and Clinical Research Institute, Newcastle University, Newcastle upon Tyne, UK; c PRISM Research Group, Lifespan and Population Health, School of Medicine, University of Nottingham, Nottingham, UK; d Department of Respiratory Sciences, University of Leicester, Leicester, UK; e Development Centre for Population Health, University of Leicester, Leicester, UK; f Faculty of Health and Life Sciences, Institute for Allied Health Sciences Research, De Montfort University, Leicester, UK; g Dementia Centre of Excellence, enAble Institute, Curtin University, Bentley, Australia; h Institute of Mental Health, University of Nottingham, Nottingham, UK; i Cambridge Public Health, University of Cambridge, Cambridge, UK

**Keywords:** Alzheimer’s disease, attitudes, dementia, ethnic minorities, low socioeconomic status, preferences, screening

## Abstract

**Background:**

Increased understanding of dementia risk-reduction and early detection of Alzheimer’s disease and related disorders has spurred interest in the identification of risks for dementia, underlying putative biologies, or dementia itself. Implementation of such approaches require acceptability to the public. Research prior to 2012 indicated limited acceptability for population dementia screening. The changing landscape of dementia prevention research may influence recent perceptions. Additionally, perspectives from underserved populations, such as ethnic minorities and low socio-economic groups, are lacking.

**Objective:**

In this systematic review, we sought published studies since 2012 on attitudes and preferences of people with dementia, carers and the general public from ethnic minorities and low socio-economic groups regarding dementia screening.

**Methods:**

This review was preregistered on PROSPERO (CRD42023384115) and followed PRISMA guidelines. Key search terms were entered into five databases. Articles were included if they focused on population or risk screening for dementia via primary/community care-based assessments, and which included majority ethnic minority or low socio-economic groups or discretely considered these groups in data analysis. Data were synthesized narratively.

**Results:**

Seven studies reported perspectives of ethnic minorities regarding dementia screening; one study included people from low socio-economic groups. Results indicated that participants from ethnic minorities were willing to undergo dementia screening. Predictors of willingness included belief in benefits, desire to boost diversity, and to implement lifestyle changes. Unwillingness was associated with anxiety regarding results.

**Conclusions:**

Although there seems to be high acceptability for screening in the studied groups, more research is necessary to explore the practical considerations for screening such as cultural and economic barriers, trust, and post-screening actions.

## INTRODUCTION

Dementia is a progressive syndrome, with many underlying potential causes, characterized by cognitive deficits leading to functional impairment.^1^ Globally, approximately 55 million people were estimated to be living with dementia in 2019,^2^ with numbers predicted to increase to 139 million by 2050. In addition to increasing age, the identification of modifiable risk factors for dementia, including smoking and excessive alcohol consumption, levels of physical, cognitive and social activity, hearing loss and preventing and managing chronic conditions such as diabetes and hypertension, has led to the World Health Organization^1^ classifying dementia as a global public health priority. Analysis of these modifiable risk factors has led to estimates that 40% of future dementia cases could be preventable.^3^

From a policy perspective, there has been longstanding emphasis on earlier, more timely diagnosis to allow people with dementia and their families the option to undertake future shared decision-making.^1,2,4^ Earlier detection may be approached in several ways. At a pre-symptomatic stage, systemic population screening involves identifying an illness, such as dementia, amongst a population of apparently asymptomatic individuals.^5^ This form of population screening is currently not recommended for primary care,^6–9^ as it does not meet international criteria for adoption of screening programmes.^10^ One requirement of this criteria is that the test is acceptable to the public. Another option for pre-symptomatic identification of an illness is targeted case finding, where individuals at known high risk of developing dementia for example, those with multiple risk factors, are assessed at an asymptomatic/undetected phase; however the acceptability, effectiveness, and cost effectiveness of this approach remains undetermined.^11^

The WHO classification of dementia as a public health condition has resulted in greater attention being paid to targeted, and individualized dementia risk reduction approaches as a means of reducing future dementia burden. A largescale international consortium is testing individualized approaches to dementia risk reduction (International FINGERS). Research studies and surveys carried out by charitable organizations have captured positive attitudes regarding dementia screening from the public. In the USA, 75% of older adults expressed willingness to take a test to predict their likelihood of developing dementia in the future.^12^ Similarly, evidence from a survey of 2,106 adults in the UK, with 51% indicating previous lived experience, caring responsibilities or connections with dementia, found 74% would be interested in knowing if they were at higher risk of developing dementia before symptoms occur, suggesting that dementia risk screening and population screening could be acceptable to many, should the evidence base justifying it be strong enough.^13^ However, it must be noted that these research methods rarely provide the public with empirical evidence on the risk or benefits of screening, and results should be interpretated with caution.

In terms of potential screening tests for earlier detection of dementia, a systematic review, considering the evidence base up to 2012, suggested that simple, non-invasive assessment methods such as pen and paper cognitive assessments, blood-based biomarkers and telehealth assessments which could be used in primary care, would be most suitable.^5^ The review also concluded that acceptability of screening is a complex and multi-factorial issue that is mainly deemed unacceptable to the public. At that time, seven factors contributed to the public’s perception of acceptability, including lifestyle and life view, dementia awareness, role of clinician, existing health state, benefit, communication and role of family. However, this review was unable to make any statements relevant to under-served populations, i.e., demographic groups with typically low inclusion rates in research, higher healthcare burdens or which require better service provision from research/healthcare.^14^ Ethnic minority groups are significantly under-served in dementia research, as they are under-represented in clinical trials and dementia prevention programmes^15^ which means that clinical and policy decisions may not reflect discrete cultural and social needs.^16^ Additionally, these groups have unique barriers and experiences which may negatively impact help-seeking behaviors regarding cognitive concerns, and current cognitive assessments may not be culturally-sensitive or appropriate, leading to further inequities in their access to diagnostic services.^17–19^ Additionally, socio-economic status is generally under-reported in dementia research despite lower socio-economic groups having a higher dementia risk^20,21^ and worse prognostic outcomes following diagnosis.^22^ To ensure information relevant to policy decisions regarding implementation of dementia screening is representative, it is important to consider these key under-served groups’ unique perspectives on the acceptability of dementia screening, and the facilitators and barriers for undergoing such screening. For example, in relation to other healthcare screening services, people from ethnic minorities highlighted cultural factors, religion and acculturation as key influences on uptake,^23–25^ while those from lower socio-economic groups have lower uptakes of cancer screening services.^26^ Given the rapid knowledge changes in the dementia risk-reduction and treatment landscape, and the greater emphasis on including under-served populations in research, the aim of this systematic review is to update the Martin et al.^5^ review with a specific aim to evaluate the attitudes and preferences of people affected by dementia, carers and the general public from ethnic minorities and low socio-economic groups regarding screening for dementia; both perceptions on targeted case finding and general population screening for undetected dementia are included. This review will update researchers and policy-makers focused on developing dementia screening initiatives on the current status of acceptability of screening programs, key to international criteria for implementation.^7,8,10,27^ Additionally, findings from this review will inform recommendations for future dementia screening initiatives, based on the perspectives and priorities of these key under-served groups.

## METHODS

This systematic review was preregistered on PROSPERO (CRD42023384115) and reporting follows PRISMA (Preferred Reporting Items of Systematic Reviews and Meta-Analyses) guidelines (see the Supplementary Material).

### Search strategy

The search strategy (see the Supplementary Material) was developed using Martin et al.’s search strategy as a foundation.^5^ This included search elements capturing 1) dementia, 2) screening and case finding, and 3) attitudes and preferences. Additional elements were added to yield results specific to 4) ethnic minority groups and 5) low socioeconomic groups. The updated search strategy was developed collaboratively by the research team with the guidance of a librarian at Newcastle University and the ‘Peer Review of Electronic Search Strategies’ (PRESS) guidelines.^28^

Six databases were search electronically; MEDLINE, EMBASE, PsycINFO, CINAHL, CDR, and Cochrane Library in February 2023 (see the Supplementary Material for EMBASE example). Search results were restricted to studies published between August 2012 and February 2023 to develop upon Martin et al.^5^ previous systematic review.

### Inclusion and exclusion criteria

The current review followed similar criteria to Martin et al.^5^ with a specific focus on ethnic minorities and low socio-economic groups. It included papers whose primary or secondary objective was to explore the attitudes and preferences of the public, people living with dementia, carers and health and social care professionals towards population screening for dementia. Table 1 shows the full eligibility criteria for this review.

**Table 1:** Eligibility criteria for article selection

Factors	Inclusion criteria	Exclusion criteria
Language	All languages	n.a
Time frame	Published in August 2012	Published prior to August 2012
Location / setting	Any location or setting	n.a
Topic	Population screening for dementia.	- Attitudes towards population screening for mild cognitive impairment (who do not meet the criteria of dementia) as this is a clinically contentious label^54^
		- Attitudes towards early or timely diagnosis. These concepts relate to the identification of prodromal or clinical dementia when the individual is starting to present or is fully presenting symptoms^55^
Intervention / Exposure	Tests that can currently be easily administrated and using variables that can be easily ascertained in primary or community care settings to screen for dementia e.g., electronic, pen and paper, biomarkers.	- Any tests that cannot be easily carried out in primary care, e.g., genetic tests, lumbar puncture, Amyloid PET imaging
		- Screening tools to detect persons with mild cognitive impairment
Comparator / Control	Any comparator or no comparator	n.a
Population	Members of the general public, people living with dementia and carers (informal or formal). All irrespective of age and education but must involve a majority ethnic minority or low-socioeconomic group, or discretely consider ethnic minority and socio-economic groups in their data analysis approach.	- Studies using samples from the general public, people living with dementia and carers (informal and formal) that do not include a majority ethnic minority or low socio-economic group, or which do not consider the ethnic minority and socio-economic groups discretely in their data analysis approach.
Study type	All study types (qualitative, quantitative and mixed methods)	Case studies
Publication type	Peer reviewed publications	- Unpublished sources
		- Opinion based papers
		- Conference abstracts
		- Reviews

Importantly, three key definitions were used to define our topic and population groups of interest and to inform our eligibility criteria. Population screening was defined as the testing of individuals who have previously not sought help for dementia related symptoms and are not under active surveillance (e.g., diagnosed with mild cognitive impairment) including asymptomatic individuals, for any types of dementia.^5^ Ethnic minority groups were defined as “a group of people from a particular culture or of a particular race living in a country where the main group is of a different culture or race”.^29^ Low socioeconomic groups were defined as “the position of an individual or group on the socioeconomic scale, which is determined by a combination of social and economic factors such as income, amount and kind of education, type and prestige of occupation, place of residence, and—in some societies or parts of society—ethnic origin or religious background”.^30^ Low socioeconomic status (SES) measures need to either explicitly state how they are using one of the above variables as a proxy for overall SES or provide a consideration for multiple of the above variables in contributing to low SES.

### Selection process

All citations were exported to Rayyan, where titles were screened by the first reviewer (MB), whilst a second reviewer (MH) independently screened a random sample of 40% of all titles. Disagreements were recorded and resolved via discussion between the two reviewers. Both reviewers screened all remaining abstracts. Discrepancies were resolved by a third reviewer (AH) via discussion. The full texts of articles identified as either relevant or possibly relevant from the abstract were obtained and assessed to determine whether they met the inclusion criteria by two reviewers independently (MB and AH). Discrepancies between reviewers were resolved via discussion with a third reviewer (RMA). Reasons for exclusions at full text were recorded. A hand search of reference lists of included papers and snowball sampling of any possibly relevant reviews generated by the search was conducted to identify additional relevant articles.

As population screening for dementia can be discussed as part of a general diagnostic process, papers related to dementia diagnosis were included to full text stage to ascertain if any references to population screening were made. Similarly, as demographic information may not be cited until the results section of many publications, papers that did not mention any reference to ethnic minorities or low socioeconomic status were also included to full text stage.

Authors of studies were also contacted if there were issues obtaining or interpreting data. This was primarily regarding the eligibility of the study where the demographic information or outcomes of interest were unclear, but authors were also contacted to clarify discrepancies in reported information (e.g., mismatched participant numbers between text and table) as noted in Table 2.

**Table 2:** Demographic data and key results from included studies

Name	Location	Demographics	Group membership	Study type	Study methods	Main relevant results
Erickson et al., 2022^37^	Wisconsin, USA	*N* = 334 Black participants: 148 White participants: 186 Age: Black: 64.9±8.4 years White: 64.7±7.0 Sex (female): Black: 107 (72.3%) White: 141 (75.8%) Education (with≥bachelors degree): Black: 67 (45.6%) White: 129 (69.4%) All participants were cognitively unimpaired	Ethnic minority (comparison between Black and White participants)	Mixed methods	**Quantitative:** -Alzheimer’s biomarker survey -Research attitudes questionnaire -Early onset dementia day-to-day unfair treatment -5 point Likert scale on modifiable dementia risk and concern about developing dementia, and willingness to enroll in biomarker researcher. **Qualitative:** -Open ended questions in response to five vignettes describing hypothetical biomarker studies for dementia involving disclosure/non-disclosure/PET/CSF and blood tests.	**Quantitative:** No significant differences between Black and White groups for willingness to enroll in biomarker research studies. **Qualitative:** ***Willingness to enroll in dementia biomarker studies*** (Themes) -Personal interest -Supporting research ***Unwillingness to enroll in dementia biomarker studies*** -Anxiety -Limited utility of testing -Physical harms of testing -Burden of Testing -Stigma
Fowler et al., 2012^38^	Indiana, USA	*N* = 554 African American: 56.5% White: 41.5% Other: 2% Age: 65.5% were ≥70 years old Sex: 70% female Education: 42.1% had less than a high school education	Ethnic minority (comparisons between racial/ethnic minority and White participants)	Quantitative	PRISM-PC – a Likert scale to measure attitudes on acceptability, benefits and harms of dementia screening.	No differences in willingness to undergo screening found for ethnicity – high willingness (89.5% African American, 87.5% “Other”, 90.4% White willing to accept screening). Those who were willing to undergo screening demonstrated significant differences in better perception of benefits, benefits in future healthcare planning, benefits in screening for other conditions, and greater belief that a treatment for Alzheimer’s disease would be available compared to those who were unwilling. Odds ratio scores for refusing screening were higher in 70–74-year-olds (OR: 5.65), 76–79-year-olds (OR: 3.63), than 65–69-year-olds, and lower for those with higher beliefs in the benefits of screening (OR: 0.85).
Galvin et al., 2020^39^	New York, USA	*N* = 288 66.9% White 25.9% African American 47.7% Hispanic ethnicity (comprised of 36.1% South American, 33.1% Puerto Rican, 11.3% Dominican, 10.6% Mexican and Central American; 3.8% Cuban, and 5.3% Other/Not Specified). 70.5% female Mean age: 71.52±8.3 y (range: 55–100) Education: 13.3±4.8 y (range: 0–20). Hollingshead Index of Social Status was 40.8±19.1 (range 11–77)	Ethnic minority (considers ethnicity within analysis)	Quantitative	10 question interview following dementia screening: 5 questions regarding satisfaction with the information received, measured via a Likert Scale. 5 questions regarding adherence with recommendations to share results with HCPs/family members, and make lifestyle changes, measured via yes/no response and open ended questions (answers categorized).	92.7% participants had a positive experience with the screening program. No preferences between type of dementia screening task (interview vs pen and paper) by sex, race, ethnicity or SES (results not shown). 56% reported sharing results with family following dementia screening; race and ethnicity were not predictors in the final model. 32% shared screening results with HCPs; this did not vary by age, sex, race or ethnicity. 49% reported a change in behavior following dementia screening; race and ethnicity were not predictors in the final model.
Grigsby et al., 2017^35^	Los Angeles, USA	*N* = 42 90% Hispanic/Latino 83% female Mean age = 42.2 years (SD = 11, range = 24 to 73 years) 41.5% with less than a high school education	Ethnic minority (majority racial/ethnic minority)	Mixed methods	Participants were part of an intervention where they were exposed to an audio tele-novella about dementia and qualitative and quantitative approaches were explored afterwards. **Qualitative:** Four focus groups **Quantitative:** Questionnaire on dementia attitudes and knowledge, employing a 5 part Likert scale.	**Qualitative:** Overall belief with participants that knowing their dementia risk would allow them to slow down progress and live a normal life for longer. **Quantitative:** Participants showed a high belief in benefits for prognosis following the telenovela (mean 4/5 for “Early detection of dementia will make it easier to manage and treat it”) and a showed a high willingness for dementia screening (mean 4/5 for “I would like to know if I’m at a higher risk than others for developing dementia”).
Ludecke et al., 2016^40^	Germany	*N* = 1, 795 Socioeconomic status: 24 % low, 61 % medium, 15 % high Female: 51.8% Age: 18–79 years 33% had contact with a person with dementia. 12.3% were caring for a person with dementia.	High, middle and low SES SES index was calculated by summing up the values of the three indicators, resulting in a scale ranging from 3 to 27 points. Based on this index, three status groups representing low (3–10), middle (11–19) and high SES (20–27) were defined.	Quantitative	Adapted Alzheimer’s disease knowledge scale (ADKS) 3 items investigating attitudes/beliefs about dementia.	Low SES more skeptical about the benefits of knowing about their dementia risk/early dementia detection (i.e., middle and high SES were 64-51% respectively less likely to agree with the statement “to know early that you have dementia is not good at all”).
Neugroschl et al., 2019^33^	New York, USA	*N* = 33 (focus groups), 49 (survey) Latino: 100% Mean age: Focus groups: 70.67 y Survey: 75 y	Ethnic minority (racial/ethnic minority solely)	Mixed methods	Participants were involved in focus groups to co-develop an educational video to raise dementia awareness. Then, different participants participated in a follow-up survey after watching the video. **Qualitative:** Six focus groups **Quantitative:** 3 item survey about	**Qualitative:** Participants associated the word “dementia” with “craziness” and associated memory screening with “electroconvulsive therapy”, fearing they would have their “heads wired” or having “cables en la cabeza” (cables in the head) as part of the evaluation. **Quantitative:** 80% of participants were willing to receive screening following a targeted health education intervention.
Palazzo et al., 2021^34^	Washington, USA	*N* = 40 **Ethnicity** Non-Hispanic White: 41% Black/African American: 21% Asian-American: 15% Hispanic/Latino: 5% Other race or multiple races: 19% Age: 3% 55–65 y 23% 65–74 65% 75–84 y 10% 85+ y Sex: 63% female 10% With dementia 23% with MCI 15% caregivers. 53% No diagnosis/association with dementia	Ethnic minority (majority racial/ethnic minority)	Qualitative	Five focus groups	Overall, participants were willing to know about their dementia risk. ***Benefits to screening:*** Allow people to engage in future planning while cognition is intact, improve their own and their family’s ability to deal with new challenges, implement lifestyle changes to enhance patient health and wellbeing, allow families to prepare for caring responsibilities and be prepared for behavioral changes (improving social support). Could slow progression or improve prognosis. ***Negative perceptions of screening:*** stress, anxiety, social isolation, knowing people who had a poor prognosis and few therapeutic options. Stigma could damage social interactions. False positives, could conflate a “risk score” with a diagnosis.
Kirk Wiese et al., 2019^36^	Florida, USA	*N* = 21 76% ethnic minority, composed of Hispanic American, Native American, African American, Afro Caribbean* Age: 58.2 y (mean) Sex Female (16) Male (5) Education: 14.4 y (mean)	Ethnic minority (majority racial/ethnic minority)	Mixed methods	**Qualitative:** -Open ended, semi-structured interview questions, exploring perceptions of cognitive screening **Quantitative:** -PRISM-PC (perceptions regarding investigational screening for memory in primary care) instrument -Health literacy assessment measured via the rapid estimate of literacy in medicine, short form	**Qualitative** The researcher/educator engaging in cognitive screening must be from the community or have engaged sufficiently to be trusted by the community. **Quantitative** 81% would want an examination annually to determine if they developed memory problems or AD. 85% would want to know they were at higher risk than others for developing it. 100% believed that earlier screening could provide opportunity for improved treatment. 86% felt family could provide better care if they know earlier. 100% believed they would have more time to plan for the future. Ethnicity did not predict willingness to undergo dementia screening.

*Discrepancies noted in original paper for prevalence of ethnicities between reporting in text and tables – figures reported were clarified with the corresponding author. MCI = mild cognitive impairment, HCP = healthcare professionals, PRISM-PC = Perceptions Regarding Investigational Screening for Memory in Primary Care.

### Data extraction

Data extraction forms developed by Martin et al.^5^ were piloted on a small number of papers and amended by MB to include explicit reference to ethnicity and socioeconomic status in the review’s primary and secondary outcomes. Our primary outcomes included perceptions, views, attitudes, preferences or experiences of people living with dementia and cognitive impairment, carers and members of the general public from ethnic minority and low socioeconomic groups, particularly 1) their experience of discussing, under-going or receiving the results of a screening test, 2) their view of population screening as an intervention (positive, negative), with quotes in support of views and perspectives, and 3) quantitative data reflecting preferences. Secondary outcomes included ethical, moral and cultural issues; practical implications in terms of knowledge, organization of health and social care (e.g., accessibility of services, information and support), resources and funding in the context of the perception of patients, carers and practitioners.

The key measures for interest for data extraction were 1) demographic information, 2) group membership (ethnic minority/low socio-economic group), 3) study methods (quantitative, qualitative, mixed methods), 4) participants’ experiences of screening, and 5) our aforementioned primary and secondary outcomes. Data from all included articles were extracted into an Excel form by one reviewer (MB). The data extraction form was reviewed independently against the included articles by a second reviewer (RMA) and discrepancies were discussed, addressed and amended.

### Quality assessment

To account for the broad range of study designs used in this area of health care, the ‘Mixed Methods Appraisal Tool’ (MMAT)^31^ was used independently by two reviewers (MB and AH) to assess quality of all included papers. The MMAT appraises the methodological quality of five types of study design: qualitative, quantitative descriptive, mixed methods, randomized controlled trials, and non-randomized studies. Discrepancies were resolved via discussion. Due to an estimated paucity of literature, all studies were included regardless of their quality level (see Supplementary Table 1).

### Data synthesis

Narrative data synthesis was conducted to answer our research aims. This considered the primary and secondary outcomes of our review. Quantitative and qualitative data were integrated according to a convergent integrated approach, according to JBI methodology for mixed methods reviews.^32^ As per the JBI methodology,^32^ quantitative data was “qualitized”, which involves translating extracted data into “textual descriptions” to allow integration with qualitative data. Codifying quantitative data as a narrative interpretation of quantitative results is considered less error-prone than adding numerical descriptors to qualitative data.^32^ Textual descriptions were pooled with the extracted qualitative data, and all data was considered and discussed by members of the research team (MB, RMA) to identify categories of similarity based on meaning. Categories were aggregated to produce the overall review findings.

## RESULTS

### Search yield

**Fig. 1:**
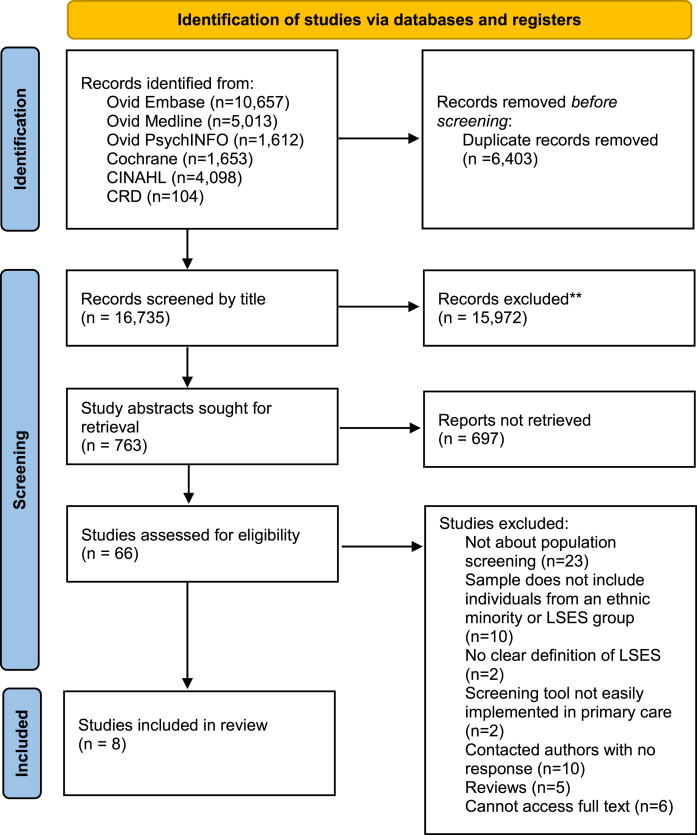
PRISMA flow chart demonstrating search yields through different phases of the systematic review. LSES, Lower socio-economic status.

The search strategy was conducted in February 2023 and generated 23,137 articles, of which 6,402 were removed due to duplication. Following title and abstract screening, 65 full texts were assessed for eligibility, of which 8 were included as seen in Fig. 1.

### Demographic information

The characteristics of the eight eligible studies are summarized in Table 2. All articles were published between 2012–2022. Seven studies included racial/ethnic minorities (Racial/ethnic minority solely (*n* = 1),^33^ majority racial/ethnic minority (*n* = 3),^34–36^ comparisons between racial/ethnic minority and White participants (*n* = 2),^37,38^ or considers ethnicity discretely within analysis (*n* = 1)^39^). One study included participants with low socio-economic status (compared to middle and high SES).^40^ Studies employed quantitative analysis (*n* = 3),^38–40^ qualitative (utilizing focus groups; *n* = 1),^34^ and mixed methods (using Likert point surveys such as the Perceptions Regarding Investigational Screening for Memory in Primary Care (PRISM-PC) with either semi structured interviews, focus groups or vignettes; *n* = 4).^33,35–37^

Countries represented in this review include the USA (*n* = 7)^33–39^ and Germany (*n* = 1).^40^ Two studies involved interventions where attitudes were measured post viewing of educational materials, i.e., audio and visual materials regarding factual information about dementia symptoms, impact, and cognitive screening.^33,35^ Two studies involved administrating simple dementia screening tests,^38,39^ while one involved assessing enrolment into hypothetical dementia biomarker studies.^37^ Regarding type of screening, one study evaluated perspectives on screening presence of dementia biomarkers for research purposes,^37^ two studies considered perspectives on a combination of dementia risk screening or screening for earlier detection of dementia^35,36^ and five studies considered some form of cognitive screening for undetected dementia.^33–35,38–40^

Sample sizes ranged from 21 to 1,795. Participants included cognitively intact adults of any age,^35,36,40^ adults aged ≥45 years,^37,39^ older adults (≥65),^33,38^ people with a diagnosis of mild cognitive impairment or dementia,^34^ and carers.^34,36^ Ethnicities represented in the sample included African-American ^34,36–39^, African Caribbean,^36^ Hispanic,^33–36,39^ Asian,^34^ White,^34,37–40^ Native American,^36^ and other or multiple races.^34,38^ Socioeconomic statuses represented in the one study that considered them were high (15% of sample), medium (61% of sample) and low (24% of sample).^40^ Overall quality of studies was high, with more information provided in Supplementary Table 1.

### Attitudes and preferences of people in racial/ethnic minority and low socio-economic groups

#### Awareness of dementia

Studies highlighting issues surrounding awareness of dementia considered perspectives from Hispanic^33,35^ and lower socio-economic groups.^40^ Knowledge of dementia was found to be poor in Hispanic participants but improved markedly after exposure to educational materials.^33,35^ For example, there was a misconception that dementia signifies “craziness”, and a fear that memory evaluations would involve procedures akin to electroconvulsive therapy.^33^ Another study in Germany suggested socioeconomic status is a variable associated with dementia knowledge, with higher socio-economic groups showing more awareness of treatment, prevention and diagnosis.^40^

#### Attitudes and willingness to undergo dementia screening

Studies considering people’s attitudes and willingness to be screened for dementia were concerned with racial/ethnic minority groups including Hispanic (*n* = 2), Black/African American (*n* = 2) and groups representing multiple ethnicities (*n* = 2),^33–38^ while a German study considered those with low socioeconomic status.^40^

Attitudes and willingness to undergo dementia screening was assessed in the following ways: using the PRISM-PC questionnaire (*n* = 2),^36^ through a 4-part Likert scale question “to know early that you have dementia is not good at all” (do not agree at all – strongly disagree; *n* = 1),^38,40^ through qualitative interviews following an education intervention regarding the symptoms of dementia and the impact it has on families (*n* = 1),^35^ through an anonymous questionnaire asking if participants would like to undergo cognitive screening following an education video regarding memory assessments (*n* = 1),^33^ through qualitative interviews regarding preferences for timing of dementia diagnosis and acceptability of a tool to assess undiagnosed dementia risk (*n* = 1),^34^ and by ranking likeliness to enroll in a biomarker study based on five possible scenarios (with results disclosure, without results disclosure, using positron emission tomography (PET) scans, collecting cerebrospinal fluid or collecting blood samples) via a 5-point Likert scale (*n* = 1).^37^ However, responses regarding willingness are dependent on the degree of evidence available to enable participants to make informed decisions; this information was unavailable beyond brief descriptions of educational interventions in two studies.^33,35^

All studies concerning racial/ethnic minority groups indicated a high willingness to undergo dementia screening, based on the assessments outlined above. No significant differences were found between African American groups with White participants.^37,38^ People in low SES groups were more skeptical about dementia screening, and more likely to agree with the statement “to know early that you have dementia is not good at all” compared to those in middle and high SES groups.^40^

The predictors of willingness/unwillingness to undergo dementia screening were explored in African American groups.^37,38^ One study involved providing participants with five vignettes describing hypothetical dementia biomarker studies to evaluate willingness to undergo dementia screening,^37^ while the other involved asking participants to undergo a written dementia screening test following a questionnaire regarding perceptions and attitudes towards dementia screening.^38^ Both studies indicated that those who had greater beliefs in the benefits of dementia screening, such as the modifiable nature of dementia risk, were more likely to undergo screening. Qualitative data indicated that those in African American groups would undergo screening to “boost diversity” in dementia biomarker research, out of personal interest regarding their cognitive health, to implement lifestyle changes and to support research generally.^37^ Regarding unwillingness to undergo dementia screening, those who were unwilling were significantly less likely to believe there would be a treatment for dementia, and that those in the 70–79 age group were less likely to want to undergo screening.^38^ Qualitative findings also suggested that people would be less willing to be screened if they were anxious about high-risk results, concerned that they would experience stigma and discrimination from their employers and insurance companies due to a positive result, if they believed there was limited utility to testing or if it would take significant time and travel to undergo the screening test.^37^

#### Perceptions of the outcomes of dementia screening

Studies concerning racial/ethnic minorities highlighted positive perceptions towards the outcomes of dementia screening.^34–36^ All studies indicated that participants felt that knowing their risk of dementia early would improve their ability to manage and treat the condition; manner of treatment was undefined. Findings indicated that participants felt they would be able to implement lifestyle changes to slow progression and improve prognosis.^34,35^ Participants believed dementia screening could allow them to better plan for the future and improve their family’s care provision,^34,36^ and suggested that families would have more time to prepare for caring responsibilities and better understand the onset of behavioral changes, therefore enhancing social support.^34^

Negative perceptions of the outcomes of dementia screening were reported.^34,36^ Findings indicate that a positive dementia screening result could lead to psychological distress, such as anxiety and depression, and stigma from others, social isolation and psychological harm due to few therapeutic options, if the person knew people who had a poor dementia prognosis.^34^ There was also concern regarding “false positives” and the worry that people may conflate a risk score with a diagnosis.

#### Practical considerations for any implementation of a dementia screening program

Practical considerations for screening in USA-based ethnic minorities, including Hispanic (*n* = 2), African American (*n* = 2), and groups composed of multiple ethnicities (*n* = 2), identifying the following: cultural and economic barriers to screening, communication and trust, type of screening test, and post-screening actions. Barriers to screening, should such programs be evidenced and implemented, for ethnic minorities would include transportation issues, language barriers, health insurance and lack of confidence in medical care.^33,35^ Economic barriers were also recognized in another study, highlighting the potential negative impact of screening results on current employment.^36^ Screening should occur in the context of a trustworthy healthcare provider relationship to be considered acceptable,^34,36^ and the person involved in cognitive screening should either be a member of the local community or engaged enough with the community to be considered trusted.^36^ It was also considered vital that clarity is provided around the difference between identifying risk and receiving an early diagnosis as the difference may not necessarily be apparent to the person.^34^ The less invasive the screening method, the higher the willingness of the patient to partake in it would be,^37,38^ for example, pen and paper tasks are preferable to biofluid markers.^37^ Post-screening, findings indicated that only half of patients would share results with family or initiate a behavior change, and that there is a need for additional resources to provide emotional support when discussing screening results.^34,39^ Those with greater cognitive impairments following a dementia screening test were less likely to share results with families compared to those without impairments.^39^

## DISCUSSION

To our knowledge, this is the first systematic review to consider the attitudes and preferences of under-served groups, such as people from ethnic minorities and low socio-economic groups, regarding dementia screening. Most papers considered general population screening for undetected dementia, as opposed to targeted case-finding in those at high risk of dementia. Key results suggest that willingness to undergo screening is relatively high in diverse ethnic minority groups, but that there is a significant gap in the literature regarding low socio-economic groups and ethnic groups outside USA. Willingness to undergo screening is linked to perceived benefit of results, promotion of diversity within research, and negative outcomes relating to a positive result. Practical considerations for screening include awareness of cultural and economic barriers, trustworthiness of healthcare providers, post-screening outcomes and use of non-invasive tests.

Ethnic minority participants in the reviewed studies were highly willing to undergo dementia screening—both for screening of undetected dementia and for dementia risk. It is unclear what materials these individuals had to guide these decisions, and what evidence bases were presented, or what media these groups consumed. Positive perceptions of dementia screening were associated with belief that early detection would support management and treatment of dementia and decelerate progression via lifestyle changes. This is in contrast to a previous review, which included a majority white population that reported a low willingness to undergo screening (Martin et al.^5^). The differences between the previous review and ours may relate to cultural factors and/or may be reflective of the changing landscape of dementia research and risk awareness since 2015. For example, regarding screening for dementia risk, the first Lancet Commission on dementia prevention, intervention and care was published in 2017 and suggested that approximately 35% of dementia cases were attributable to modifiable risk factors^40^; the updated report in 2020 increased this to 40% preventable dementia cases.^3^ Regarding screening for undetected dementia, WHO released their global action plan for dementia in 2017, calling for the development of national policies and plans for dementia across the globe, including the promotion of early diagnosis in an unspecified manner, improvement of the general public’s knowledge about dementia and requirement of case-finding services for undetected dementia within healthcare.^1^ Given the prevalence of USA-based studies in this review, it is notable that the Alzheimer’s Association released guidance in 2013 for systematic cognitive screening during the Medicare Annual Wellness visit in those aged 65 or over, while the American Academy of Neurology recommended annual cognitive screening in this population in 2019.^41,42^ These advances were met with significant media interest and public health campaigns which may have increased the perception of benefits from dementia screening,^40^ despite a recent study demonstrating that dementia screening does not improve quality of life, healthcare utilization or advanced care planning.^43^

Results indicated that lower socio-economic groups were more skeptical regarding dementia screening; this is based only on one study and is therefore not generalizable. It is notable however that there is a dearth of research considering the perceptions of those from lower socio-economic groups. Additionally, the ethnic minorities represented in this review are mainly drawn from USA studies and therefore findings on attitudes and preferences may not be transferable to those living in other regions. Additionally, while ethnic groups were often described in broad categories (e.g., Hispanic), there can be significant heterogeneity within these groups, as noted by Galvin et al.^39^, who encompassed South American, Puerto Rican, Dominican, Mexican, and Central American in their “Hispanic” category. It is likely that there are different languages, dialects, cultures, customs, religions and experiences both between and within these groups which may impact their perceptions of dementia screening; these nuances are likely not captured in the results of this review. Exploring the views of ethnic minority and low socio-economic groups across multiple high-income countries may have highlighted unique preferences regarding dementia screening, such as areas relating to cultural expectations and roles and religiousbeliefs.^44,45^

Commonalities were found between the thematic results from the systematic review by Martin et al.^5^ and this update, particularly regarding dementia awareness, the role of the clinician, communication and perceived benefits of dementia screening. Both reviews highlighted a low level of dementia awareness leading to misunderstandings regarding dementia screening; for example, members of the Hispanic population were concerned that screening would involve processes similar to electroconvulsive therapies.^33^ Culturally-specific educational programs appeared to have a positive effect on enhancing dementia awareness and increasing interest in screening in Hispanic populations.^33,35^ Martin et al.^5^ suggested that clinicians needed to communicate their role clearly and explain how they would conduct the tests and interpret results. In this updated review, ethnic minority groups were more concerned regarding the trustworthiness of the healthcare provider conducting the screening, particularly regarding their relationship in the local communities.^34,36^ Both reviews highlight similar perceptions regarding the benefits of dementia screening. However, this update suggests that members of the African American community may be more likely to become involved in biomarker studies to improve diversity within research,^37^ it is notable that this group was well-educated (45.5% with a Bachelor’s degree). Specific cultural and economic barriers to screening were also highlighted in this review regarding language issues, lack of transport, lack of confidence in medical care and worries regarding impact on health insurance and job security.^33,35,36^

In terms of the implications for public health policy and practice, the 2023 World Alzheimer Report, *Reducing Dementia Risk; Never too early, never too late*’ concluded that “in the absence of a cure or a treatment that is globally accessible, risk reduction remains the most feasible and proactive way to combat dementia” on an international scale.^2^ It urged governments to include national risk reduction programs within their dementia plans/strategies and invest more research funding into this area, in addition to the search for new treatments; the latter are highly unlikely to be readily available to the majority of people with dementia worldwide who live in Low-Middle Income Countries (LMICs). Additionally, national guidelines (e.g., 5th Canadian Consensus Conference on the diagnosis and treatment of dementia) have suggested that screening for undetected dementia may be appropriate when patients are identified with elevated risk, as per recognized risk factors.^46^ This review has shone a light on the attitudes and preferences of ethnic minorities on dementia screening; however, the findings are based on research from two High Income Countries, with a significant absence of empirical research from LMICs. This is a significant research gap as some LMICs such as South East Asia countries, and minority groups, e.g., those of South Asian ancestry, are predicted to experience the highest future increases in dementia numbers in the next few decades.^47^

Ethnic minority groups have different risk factors, prognosis, health seeking behaviors, and experiences of care, marginalization and systemic racism compared to non-ethnic minorities. Regarding dementia, specific cultural attitudes and disease-related stigma have been described within ethnic groups and national risk reduction strategies would need to be inclusive of such societal attitudes and beliefs.^4^ Thus under-representation of ethnic minorities’ perspectives in dementia research leads to the development of clinical and policy decisions that do not reflect their needs. This review demonstrates tangible findings regarding cultural and economic considerations for the practicalities of dementia screening. Based on these findings, we have developed recommendations as presented in Table 3.

**Table 3:** Recommendations for dementia screening, considerate to ethnic minority populations

Area of Interest	Recommendations
Dementia Awareness and Education	•Information about dementia, dementia risk-reduction and prevention, and dementia screening should be conveyed in a culturally-appropriate and meaningful way, such as through common channels of communication for different ethnic groups, e.g., the Latino cable channel “tele-novellas” in the USA. This may increase the acceptability of dementia screening and improve help-seeking in different populations.
Cultural and Economic Barriers for screening	•Dementia screening tools should be non-invasive. •Information regarding dementia screening and dementia screening tools should be translated into multiple languages, and appropriate translators should be made available. •Information regarding the dissemination and disclosure of results must be clearly stated, such as who results will be disclosed to and how this might impact important services, e.g., insurance.
Communication and Trust	•Trusted community advocates may be beneficial to navigate the dementia screening process in under-served groups. These could support healthcare practitioners to approach these groups and convey information in a culturally-sensitive and appropriate manner. •The difference between dementia risk and dementia diagnosis must be clarified to reduce undue distress on patients and family members.
Post-Screening Actions	•Post-screening pathways must be clearly communicated, such as who to speak to about results (i.e., healthcare practitioners, family members) and which services can provide emotional and behavioral change support.

Significant strengths of this systematic review include building upon the previous Martin et al.^5^ review with consideration of the changing landscape regarding inclusion of under-served populations in research.^14^ This has important implications as different sociodemographic groups may respond differently to the offer of dementia screening and risk-reduction, and interventions cannot be appropriately tailored without synthesizing the research within these groups. This review’s protocol was pre-registered, and PRIMSA guidelines were followed throughout the process. Additionally, any future studies in this area must ensure that the knowledge base of the individuals responding with regard to the actual state of evidence regarding ‘early’ diagnosis is known in order to interpret the findings. Opinions can change when full disclosure of the gaps in evidence are fully described. For example, participants in a citizen jury study did not believe GPs should practice case-finding for dementia after expert information regarding benefits, challenges and harms were presented to them.^48^

However, due to limited resources, our title screening involved one reviewer screening all titles and a second reviewer screening a random selection of 40% of the titles. While this provides a quality control mechanism to minimize bias, and is more reliable than a single screening process, this may have affected our ability to identify all relevant articles. Single screening of titles and abstracts can be considered as an appropriate methodological shortcut,^49,50^ in our case, we applied partial dual-screening to minimize error. We also note that of the 6,766 titles reviewed by both reviewers,>99% of decisions were in agreement (0.006% disagreement). The high concordance of double screening makes this unlikely.^49^ To mitigate against further risk of bias, two reviewers independently screened all abstracts and full texts, and a hand-search of the reference lists of all included texts was conducted. Additionally, although we conducted the search strategy in six databases, all articles identified were in English; alternative databases may have been more inclusive to non-English research articles, which may have impacted this review’s key results. However, it is noted that language restrictions in systematic reviews in the medical sciences field is considered to have minimal impact on results and conclusions for most topics, and is considered a viable approach for systematic reviews with limited resources.^51–53^ The majority of studies included in this review were conducted in the USA, highlighting a significant gap in the literature regarding diversity of research in non-USA countries. Additionally, only one German study considering low SES was identified, strengthening the suggestion that this group is under-served by research. However, it must be noted that we only included studies which explicitly stated they were studying SES. The definition of SES is complex and unstandardized, generally involving multiple intersectional factors such as income, education, class, place of residence, and in some cultures, ethnicity or race. Although these may be considered as “surrogates” of SES, this is not consistent across the literature. Therefore, we excluded studies that only reported findings for a discrete factor which contributes to SES (e.g., education, income), and suggest that future research consider measures to explicitly characterize SES as a composite, mindful of appropriate cultural standards, such as through national government statistics. Future work should consider greater geographical scope and inclusion of SES measures to develop a more representative understanding of attitudes and preferences towards dementia screening, in order to meet this criterion in the international screening criteria.^27^

### Conclusion

To our knowledge, this is the first systematic review to consider the attitudes and preferences of two key under-served population groups, namely ethnic minorities and low socio-economic groups, regarding dementia screening, both at population level and those at higher risk. Overall, willingness to undergo dementia screening is high in the ethnic minority groups represented in the limited number of studies identified; only one study considered low socio-economic status and so results in this group cannot be concluded. Culturally and economically-specific perceptions were reported regarding willingness to undergo dementia screening, with six practical considerations highlighted. Based on results, key recommendations are made regarding dementia awareness and education, cultural and economic barriers to screening, communication and trust, and post-screening actions. Future research should consider including ethnic minority groups from countries beyond the USA and increasing research into the preferences of low socio-economic groups regarding dementia screening.

## AUTHOR CONTRIBUTIONS

Manjot Brar (Data curation; Formal analysis; Investigation; Methodology; Writing – original draft); Ríona Mc Ardle (Formal analysis; Methodology; Writing – original draft); Alexander Hagan (Methodology; Writing – review & editing); Amani Al-Oraibi (Writing – review & editing); Matilda Hanjari (Methodology; Writing – review & editing); Blossom Stephan (Funding acquisition; Writing – review & editing); Carol Brayne (Funding acquisition; Writing – review & editing); Louise Lafortune (Writing – review & editing); Manpreet Bains (Funding acquisition; Writing – review & editing); Nadeem Qureshi (Funding acquisition; Writing – review & editing); Louise Robinson (Conceptualization; Funding acquisition; Methodology; Writing – original draft).

## Supplementary Material

Supplementary Material: PRIMA Checklist

Supplementary Material: Search Strategy

Supplementary Table

## Data Availability

Data sharing is not applicable to this article as no datasets were generated or analyzed during this study.
