# Supplementary material for: Attitudes and Preferences Towards Screening for Dementia with a Focus on Ethnic Minority and Low Socio-Economic Groups: A Systematic Review of Research Studies Written in the English Language
Source: J Alzheimers Dis. 2024 Aug 13;100(4):1315–31. doi: 10.3233/JAD-240315 (PMC11380224; doi:10.3233/JAD-240315)
Supplement: Supplementary Material: Search Strategy [file jad-100-jad240315-s002.docx]

**Supplementary Material**

**Attitudes and Preferences Towards Screening for Dementia with a Focus on Ethnic Minority and Low Socio-Economic Groups: A Systematic Review of Research Studies Written in the English Language**

**Embase Search Strategy – Public & Patients & Carer Perceptions**

| 1 | Exp dementia/ |
| --- | --- |
| 2 | exp alzheimer disease/ |
| 3 | dement*.ti,ab |
| 4 | alzheimer*.ti,ab |
| 5 | (Cognit* AND (disord* OR impair* OR declin* OR function*)).ti,ab |
| 6 | 1 OR 2 OR 3 OR 4 OR 5 |
| 7 | exp neuropsychological test/ |
| 8 | (Neuropsych* adj3 (Test* OR assess*)).ti,ab |
| 9 | (psychologic* adj3 (test* OR assess*)).ti,ab |
| 10 | exp psychological test/ |
| 11 | exp geriatric assessment/ |
| 12 | "geriatric assess*".ti,ab |
| 13 | exp questionnaire/ |
| 14 | questionnaire*.ti,ab |
| 15 | exp self evaluation/ |
| 16 | exp self examination/ |
| 17 | (self adj3 assess*).ti,ab |
| 18 | (self adj3 exam*).ti,ab |
| 19 | exp interview/ |
| 20 | ((telephone interview*)).ti,ab |
| 21 | 7 OR 8 OR 9 OR 10 OR 11 OR 12 OR 13 OR 14 OR 15 OR 16 OR 17 OR 18 OR 19 OR 20 |
| 22 | exp mass screening/ |
| 23 | ((mass screen*)).ti,ab |
| 24 | "population screen*".ti,ab |
| 25 | screen*.ti,ab |
| 26 | detect*.ti,ab |
| 27 | exp risk assessment/ |
| 28 | “risk assessment”.ti,ab |
| 29 | Exp case finding/ |
| 30 | “case finding”.ti,ab |
| 31 | “case-finding”.ti,ab |
| 32 | 22 OR 23 OR 24 OR 25 OR 26 OR 27 OR 28 OR 29 OR 30 OR 31 |
| 33 | "3 word recall".ti,ab |
| 34 | "word recall".ti,ab |
| 35 | "7-minute screen".ti,ab |
| 36 | "7MS".ti,ab |
| 37 | "6 item cognitive impairment test".ti,ab |
| 38 | "6 CIT".ti,ab |
| 39 | "AB cognitive screen".ti,ab |
| 40 | "abbreviated mental test".ti,ab |
| 41 | "ADAS-cog".ti,ab |
| 42 | "AD8 informant interview".ti,ab |
| 43 | "animal fluency test".ti,ab |
| 44 | "brief alzheimer* screen".ti,ab |
| 45 | “brief cognitive scale”.ti,ab |
| 46 | "clinical dementia rating scale".ti,ab |
| 47 | "clinical dementia test".ti,ab |
| 48 | "community screening interview for dementia".ti,ab |
| 49 | "cognitive abilities screening instrument".ti,ab |
| 50 | "cognitive assessment screening test".ti,ab |
| 51 | "cognitive capacity screening examination".ti,ab |
| 52 | "clock drawing test".ti,ab |
| 53 | "deterioration cognitive observee".ti,ab |
| 54 | "Dem Tect".ti,ab |
| 55 | "fuld object memory evaluation".ti,ab |
| 56 | "general practitioner assessment of cognition".ti,ab |
| 57 | "GPCOG".ti,ab |
| 58 | "Hopkins verbal learning test".ti,ab |
| 59 | "HVLT".ti,ab |
| 60 | "IQCODE".ti,ab |
| 61 | "mattis dementia rating scale".ti,ab |
| 62 | "memory impairment screen".ti,ab |
| 63 | "minnesota cognitive acuity screen".ti,ab |
| 64 | "mini-cog".ti,ab |
| 65 | "mini-mental state exam*".ti,ab |
| 66 | "mmse".ti,ab |
| 67 | "modified mini-mental state exam".ti,ab |
| 68 | "3MS".ti,ab |
| 69 | "neurobehavioural cognitive status exam*".ti,ab |
| 70 | "cognistat".ti,ab |
| 71 | "quick cognitive screening test".ti,ab |
| 72 | "QCST".ti,ab |
| 73 | "rapid dementia screening test".ti,ab |
| 74 | "RDST".ti,ab |
| 75 | "repeatable battery for the assessment of neuropsychological status".ti,ab |
| 76 | "RBANS".ti,ab |
| 77 | "rowland universal dementia assessment scale".ti,ab |
| 78 | "rudas".ti,ab |
| 79 | “self-administered gerocognitive exam*”.ti,ab |
| 80 | ("self-administered" AND "SAGE").ti,ab |
| 81 | "self-administered computerized screening test for dementia".ti,ab |
| 82 | "short and sweet screening instrument".ti,ab |
| 83 | "sassi".ti,ab |
| 84 | "short cognitive performance test".ti,ab |
| 85 | "syndrome kurztest”.ti,ab |
| 86 | "six item screener".ti,ab |
| 87 | "short memory questionnaire".ti,ab |
| 88 | ("short memory questionnaire" AND "SMQ").ti,ab |
| 89 | "short orientation memory concentration test".ti,ab |
| 90 | "s-omc".ti,ab |
| 91 | "short blessed test".ti,ab |
| 92 | "short portable mental status questionnaire".ti,ab |
| 93 | "spmsq".ti,ab |
| 94 | "short test of mental status".ti,ab |
| 95 | "telephone interview of cognitive status modified".ti,ab |
| 96 | "tics-m".ti,ab |
| 97 | "trail making test".ti,ab |
| 98 | "verbal fluency categories".ti,ab |
| 99 | "WORLD test".ti,ab |
| 100 | "time and change test".ti,ab |
| 101 | "modified world test".ti,ab |
| 102 | "symptoms of dementia screener".ti,ab |
| 103 | "dementia questionnaire".ti,ab |
| 104 | /or 33 - 103 |
| 105 | exp diagnostic test/ |
| 106 | exp diagnosis/ OR exp early diagnosis/ |
| 107 | diagnos*.ti,ab |
| 108 | (routine adj3 diagnos*).ti,ab |
| 109 | /or 7 - 108 |
| 110 | exp patient/ |
| 111 | patient*.ti,ab |
| 112 | inpatient*.ti,ab |
| 113 | outpatient*.ti,ab |
| 114 | client*.ti,ab |
| 115 | user*.ti,ab |
| 116 | consumer*.ti,ab |
| 117 | exp miscellaneous named groups/ |
| 118 | person*.ti,ab |
| 119 | personal*.ti,ab |
| 120 | people*.ti,ab |
| 121 | individual*.ti,ab |
| 122 | exp caregiver/ |
| 123 | caregiver*.ti,ab |
| 124 | (care ADJ giver*).ti,ab |
| 125 | carer*.ti,ab |
| 126 | guardian*.ti,ab |
| 127 | exp FAMILY/ |
| 128 | family*.ti,ab |
| 129 | relative*.ti,ab |
| 130 | relation*.ti,ab |
| 131 | spouse*.ti,ab |
| 132 | partner*.ti,ab |
| 133 | husband*.ti,ab |
| 134 | wife*.ti,ab |
| 135 | child*.ti,ab |
| 136 | daughter*.ti,ab |
| 137 | son*.ti,ab |
| 138 | father*.ti,ab |
| 139 | mother*.ti,ab |
| 140 | brother*.ti,ab |
| 141 | sister*.ti,ab |
| 142 | sibling*.ti,ab |
| 143 | "next of kin".ti,ab |
| 144 | friend*.ti,ab |
| 145 | /or 110- 144 |
| 146 | view*.ti,ab |
| 147 | experience*.ti,ab |
| 148 | perspective*.ti,ab |
| 149 | exp perception/ OR social perception.mp. |
| 150 | perception*.ti,ab |
| 151 | satisfaction*.ti,ab |
| 152 | perceived.ti,ab |
| 153 | concern*.ti,ab |
| 154 | issue*.ti,ab |
| 155 | exp attitude/ |
| 156 | attitude*.ti,ab |
| 157 | perceiv*.ti,ab |
| 158 | belief*.ti,ab |
| 159 | aware*.ti,ab |
| 160 | understand*.ti,ab |
| 161 | concept*.ti,ab |
| 162 | knowledge*.ti,ab |
| 163 | 146 OR 147 OR 148 OR 149 OR 150 OR 151 OR 152 OR 153 OR 154 OR 155 OR 156 OR 157 OR 158 OR 159 OR 160 OR 161 OR 162 |
| 164 | exp attitude to health/ OR exp patient satisfaction/ OR exp doctor patient relationship/ |
| 165 | exp patient attitude/ |
| 166 | exp public opinion/ |
| 167 | exp satisfaction/ |
| 168 | exp patient satisfaction/ |
| 169 | exp professional-patient relationship/ OR exp human relation/ |
| 170 | exp consumer/ OR exp consumer satisfaction/ |
| 171 | exp patient education/ OR exp patient compliance/ |
| 172 | (patient AND information).ti,ab |
| 173 | ((patient* OR consumer* OR client) AND (compliance* OR participat* OR accept* OR refus*)).ti,ab |
| 174 | 164 OR 165 OR 166 OR 167 OR 168 OR 169 OR 170 OR 171 OR 172 OR 173 |
| 175 | 146 AND 163 |
| 176 | 174 OR 175 |
| 177 | 6 AND 109 AND 176 |
| 178 | 6 AND 109 AND 175 |
| 179 | 6 AND 109 AND 163 |
| 180 | exp qualitative research/ |
| 181 | exp interview/ or focus group.mp. |
| 182 | qualitative*.ti,ab |
| 183 | data collection.mp. |
| 184 | survey*.ti,ab |
| 185 | exp questionnaire/ |
| 186 | ethnographic.ti,ab |
| 187 | exp observation/ |
| 188 | observation*.ti,ab |
| 189 | (grounded adj3 theory).ti,ab |
| 190 | "life experience".ti,ab |
| 191 | phenomenologic*.ti,ab |
| 192 | narrative*.ti,ab |
| 193 | discourse*.ti,ab |
| 194 | story*.ti,ab |
| 195 | stories*.ti,ab |
| 196 | 180 OR 181 OR 182 OR 183 OR 184 OR 185 OR 186 OR 187 OR 188 OR 189 OR 190 OR 191 OR 192 OR 193 OR 194 OR 195 |
| 197 | 177 AND 196 |
| 198 | 178 AND 196 |
| 199 | 6 AND 32 AND 176 AND 196 |
| 200 | 110 OR 111 OR 112 OR 113 OR 114 OR 115 OR 116 OR 119 OR 122 OR 123 OR 124 OR 125 OR 126 OR 127 OR 128 OR 130 OR 132 |
| 201 | 6 AND 32 AND 163 AND 196 AND 200 |
| 202 | 32 OR 105 OR 106 OR 107 OR 108 |
| 203 | 6 AND 176 AND 196 AND 202 |
| 204 | 6 AND 202 AND 163 AND 196 AND 200 |
| 205 | Limit 204 to yr-”2012-current” |
| 206 | exp "ethnic or racial aspects"/ or exp cultural factor/ or exp ethnic difference/ or exp ethnicity/ or exp race/ or exp race difference/ |
| 207 | (ethnicity or (ethnic adj1 minority) or ethnic minorities or race or racial minority or racial minorities or culture or cultural minority or cultural minorities).mp. |
| 208 | exp population group/ or exp ancestry group/ or exp citizen group/ or exp ethnic group/ or exp minority group/ or exp religious group/ |
| 209 | (cultural group? or racial group? or diaspora).mp. |
| 210 | exp migrant/ or exp emigrant/ or exp forced migrant/ or exp immigrant/ or exp migrant worker/ or exp refugee/ or exp asylum seeker/ |
| 211 | (migrant? or immigrant?).mp. |
| 212 | exp cultural identity/ or exp ethnic identity/ or exp national identity/ or exp racial identity/ or exp religious identity/ |
| 213 | (Arab* or Africa* or Afro* or Asian or Bangladesh* or Black or Caribbean or China or Chinese or Gyps* or Gips* or India* or Ireland or Irish or Japan* or Pakistan* or Roma or traveller* or white or Caucasian or Sikh* or Hindu* or Muslim* or Islam* or Jew* or Hispanic or Latin or (American adj1 Native) or indigenous or aboriginal or arctic or BAME or BME or continental or oceanic or ethnoracial or multicultural or cross-cultural or transcultural or interethnic or multiethnic or multi-ethnic or multiracial or mixed race or mixed-race or nomad*).mp. |
| 214 | exp minority health/ or exp limited English proficiency/ |
| 215 | exp socioeconomics/ or exp digital divide/ or exp economic status/ or exp educational status/ or exp housing instability/ or exp income group/ or exp poverty/ or exp socioeconomic distribution/ or exp socioeconomic parameters/ or exp socioeconomic vulnerability/ |
| 216 | exp social status/ or exp income group/ or exp neet status/ or exp poverty/ or exp social background/ or exp social class/ |
| 217 | (income or working class or poverty or poor* or wealth* or LMIC or LAMIC or housing instability or housing stability or housing insecurity or housing security or literacy or illiteracy or education*).mp. |
| 218 | exp community participation/ or exp social environment/ or exp social segregation/ or exp social stratification |
| 219 | exp cultural deprivation/ or exp unemployment/ or exp financial stress/ or exp homelessness/ |
| 220 | (employment or unemployed or employed or financial or homeless*).mp. |
| 221 | exp sociodemographics/ |
| 222 | exp social inequality/ or exp economic inequality/ or exp health disparity/ or exp racial disparity/ or exp social determinants of health/ |
| 223 | (sociocultural* or socio-cultural or socioeconomic* or socio-economic or sociodemographic* or socio-demographic).mp. |
| 224 | exp high risk population/ |
| 225 | exp risk factor/ |
| 226 | (High risk or low risk or risk factor or at risk).mp. |
| 227 | exp vulnerable population/ or exp disadvantaged population/ |
| 228 | exp medically underserved/ or exp unmet medical need/ |
| 229 | (vulnerable or disadvantaged or marginalized or underserved or depriv* or disparit* or barrier* or understudied or under-represented or stigma* or bias* or adversity or disproportionate* or underprivileged or excluded or exclusion or inequality or inequalities or equality or equalities or equity or inequity or diversity or diverse or inclusion or represent* or generalis* or special).mp. |
| 230 | 206 or 207 or 208 or 209 or 210 or 211 or 212 or 213 or 214 or 215 or 216 or 217 or 218 or 219 or 220 or 221 or 222 or 223 or 224 or 225 or 226 or 227 or 228 |
| 231 | 205 and 230 |
