# Supplementary material for: Attitudes and Preferences Towards Screening for Dementia with a Focus on Ethnic Minority and Low Socio-Economic Groups: A Systematic Review of Research Studies Written in the English Language
Source: J Alzheimers Dis. 2024 Aug 13;100(4):1315–31. doi: 10.3233/JAD-240315 (PMC11380224; doi:10.3233/JAD-240315)
Supplement: Supplementary Table [file jad-100-jad240315-s003.docx]

**Supplementary Material**

**Attitudes and Preferences Towards Screening for Dementia with a Focus on Ethnic Minority and Low Socio-Economic Groups: A Systematic Review of Research Studies Written in the English Language**

**Supplementary Table 1A.** Quality scores of included studies (qualitative)

| Author (Year) | Is the qualitative approach appropriate to answer the research question? | Are the qualitative data collection methods adequate to address the research question? | Are the findings adequately derived from the data? | Is the interpretation of results sufficiently substantiated by data? | Is there coherence between qualitative data sources, collection, analysis and interpretation? |
| --- | --- | --- | --- | --- | --- |
| Pallazzo (2021) | Yes | Yes | Yes | Yes | Yes |

**Supplementary Table 2B.** Quality scores of included studies (quantitative)

| Author (Year) | Is the sampling strategy relevant to address the research question? | Is the sample representative of the target population? | Are the measurements appropriate? | Is the risk of nonresponse bias low? | Is the statistical analysis appropriate to answer the research question? |
| --- | --- | --- | --- | --- | --- |
| Fowler (2012) | Yes | No | Yes | No | Yes |
| Galvin (2020) | Yes | No | Yes | No | Yes |
| Ludecke (2016) | Yes | Yes | Yes | Yes | Yes |

**Supplementary Table 1C.** Quality scores of included studies (mixed methods)

| Author (Year) | Is there an adequate rationale for using a mixed methods design to address the research question? | Are the different components of the study effectively integrated to answer the research question? | Are the outputs of the integration of qualitative and quantitative components adequately interpreted? | Are divergences and inconsistencies between quantitative and qualitative results adequately addressed? | Do the different components of the study adhere to the quality criteria of each tradition of the methods involved? |
| --- | --- | --- | --- | --- | --- |
| Wiese (2018) | Yes | Yes | Yes | Yes | Yes |
| Erickson (2022) | Yes | Yes | Yes | Yes | Yes |
| Nuegroschl (2019) | Yes | Yes | Yes | Yes | Yes |
| Grigsby (2017) | Yes | Yes | Yes | Yes | Yes |
